# High-throughput screening of tick-borne pathogens in Europe

**DOI:** 10.3389/fcimb.2014.00103

**Published:** 2014-07-29

**Authors:** Lorraine Michelet, Sabine Delannoy, Elodie Devillers, Gérald Umhang, Anna Aspan, Mikael Juremalm, Jan Chirico, Fimme J. van der Wal, Hein Sprong, Thomas P. Boye Pihl, Kirstine Klitgaard, Rene Bødker, Patrick Fach, Sara Moutailler

**Affiliations:** ^1^UMR BIPAR, Animal Health Laboratory, ANSESMaisons-Alfort, France; ^2^IdentyPath Platform, Food Safety Laboratory, ANSESMaisons-Alfort, France; ^3^Nancy Laboratory for Rabies and Wildlife, Wildlife EcoEPIdemiology and Surveillance Unit, ANSESMalzéville, France; ^4^Department of Bacteriology, National Veterinary Institute (SVA)Uppsala, Sweden; ^5^Department of Virology, Immunobiology and Parasitology, National Veterinary Institute (SVA)Uppsala, Sweden; ^6^Department of Infection Biology, Central Veterinary Institute, Wageningen URLelystad, Netherlands; ^7^Laboratory for Zoonoses and Environmental Microbiology, National Institute for Public Health and Environment (RIVM)Bilthoven, Netherlands; ^8^National Veterinary Institute, DTUCopenhagen, Denmark

**Keywords:** tick borne diseases, molecular epidemiology, surveillance, Europe, microfluidic analyses

## Abstract

Due to increased travel, climatic, and environmental changes, the incidence of tick-borne disease in both humans and animals is increasing throughout Europe. Therefore, extended surveillance tools are desirable. To accurately screen tick-borne pathogens (TBPs), a large scale epidemiological study was conducted on 7050 *Ixodes ricinus* nymphs collected from France, Denmark, and the Netherlands using a powerful new high-throughput approach. This advanced methodology permitted the simultaneous detection of 25 bacterial, and 12 parasitic species (including; *Borrelia*, *Anaplasma*, *Ehrlichia*, *Rickettsia*, *Bartonella*, *Candidatus* Neoehrlichia, *Coxiella*, *Francisella*, *Babesia*, and *Theileria* genus) across 94 samples. We successfully determined the prevalence of expected (*Borrelia burgdorferi* sensu lato, *Anaplasma phagocytophilum*, *Rickettsia helvetica*, *Candidatus* Neoehrlichia mikurensis, *Babesia divergens*, *Babesia venatorum*), unexpected (*Borrelia miyamotoi*), and rare (*Bartonella henselae*) pathogens in the three European countries. Moreover we detected *Borrelia spielmanii*, *Borrelia miyamotoi*, *Babesia divergens*, and *Babesia venatorum* for the first time in Danish ticks. This surveillance method represents a major improvement in epidemiological studies, able to facilitate comprehensive testing of TBPs, and which can also be customized to monitor emerging diseases.

## Introduction

In Europe, ticks are the most important vectors of human and animal infectious diseases, and transmit more pathogens than any other arthropod (Jongejan and Uilenberg, 2004; Colwell et al., 2011). These diseases are normally maintained in stable natural cycles involving ticks, wildlife, and/or domestic animals, whereas humans are accidental hosts (De La Fuente et al., 2008). *Ixodes ricinus* is the most widespread and abundant European tick species capable of transmitting several diseases of both medical and veterinary importance (Heyman et al., 2010). *Ixodes ricinus* has the greatest impact on human public health by transmitting Lyme borreliosis etiological agents, caused by at least four *Borrelia* genospecies in Europe: *Borrelia burgdorferi* sensu stricto, *Borrelia garinii*, *Borrelia afzelii*, and *Borrelia spielmanii*. The relapsing fever spirochete, *Borrelia miyamotoi*, is transmitted by the same *Ixodes* species and has recently been described in ticks as well as in a human case from the Netherlands (Hovius et al., 2013). In addition to *Borrelia* transmission, *Ixodes ricinus* can transmit many other pathogens, including: *Anaplasma* spp. such as *Anaplasma phagocytophilum*, *Rickettsia* spp. from the spotted fever group, *Candidatus* Neoehrlichia mikurensis, *Ehrlichia* spp., *Bartonella* spp., *Francisella tularensis*, and *Coxiella burnetii* (Parola and Raoult, 2001; Cotte et al., 2008; Fertner et al., 2012). Ticks can also transmit *Babesia* genus protozoa, such as *Babesia divergens* or the newly described *Babesia venatorum* (sp. EU1) and *Theileria* spp. (Bishop et al., 2004; Bonnet et al., 2007a).

Increased human travel, animal transport, and environmental changes are responsible for the emergence and/or spread of numerous tick-borne pathogens (TBPs) in Europe (Dantas-Torres et al., 2012). Therefore, effective tick-based surveillance is essential for monitoring human and/or animal disease emergence (Diuk-Wasser et al., 2014). Ticks harbor a variety of pathogens, some of which are obligate intracellular organisms and/or are impossible to artificially culture. Consequently, molecular approaches are thus indispensable for TBP identification. In conventional amplification-based assays, TBP detection occurs for a restricted number of target pathogens known to be transmitted by certain tick species collected at particular sites (Cotte et al., 2010). The main disadvantage of this approach is the limited number of different targets that can be tested, given the quantity of DNA required for one PCR. To improve surveillance of human and animal diseases, new investigative tools are required which perform high-throughput testing of a wider panel of TBPs.

Therefore, the aim of this study was to conduct high-throughput monitoring of tick-borne human and animal pathogens in Europe. Accordingly, we developed a novel high-throughput epidemiological surveillance method to identify both major and neglected European TBPs (bacteria and parasites). This tool utilizes a microfluidic system (BioMark™ dynamic array system, Fluidigm) that is capable of performing parallel real-time PCRs using either 96.96 chips or 48.48 chips resulting in either 9216 or 2304 individual reactions, respectively (Liu et al., 2003). In a single experiment, 94 ticks or pools of ticks can be tested for the presence of 25 bacteria and 12 parasites, as well as confirmation of the tick species. As only a few microliters of sample are required for each test, this system can also be used in conjuction with the typically low-volume DNA extracts prepared from ticks. Then we applied this method to screen 7050 *Ixodes ricinus* collected from three European countries; France, Denmark, and the Netherlands. We demonstrated increased surveillance efficiency of major and neglected TBPs, and improved monitoring of the emerging diseases important to public and animal health.

## Materials and methods

### Study area and tick collection

A total of 7050 *Ixodes ricinus* nymphs, from six different locations in France, Denmark, and the Netherlands, divided in 47 pools of 25 nymphs per site, were studied. Questing nymphs were collected using the flagging technique (Vassallo et al., 2000). In France, ticks were collected from Murbach (F1) (N 47° 55′, E 7° 9′) and Wasselonne (F2) (N 48° 37′, E 7° 27′) in 2011. In Denmark, ticks were collected from Vestskoven (D1) (N 55° 42′, E 12° 21′) and Grib Skov (D2) (N 56° 02′, E 12° 20′) in 2012. In the Netherlands, ticks were collected from the Duin en Kruidberg area (N1) (N 52° 17′, E 4° 49′) in 2010 and 2011, and from the Austerlitz area (N2) (N 52° 5′, E 5° 18′) over a period from 2008 to 2012.

### DNA extraction

Ticks were morphologically identified to species level (Pérez-Eid, 2007) and preserved at −80°C. After washing once in 70% ethanol for 5 min and twice in distilled water for 5 min, pools of 25 nymphs were crushed in 300 μl of DMEM with 10% fetal calf serum and six steel balls using the homogenizer Precellys®24 Dual (Bertin, France) at 5500 rpm for 20 s.

DNA was then extracted using the Wizard genomic DNA purification kit (Promega, France). Total DNA per sample was eluted in 50 μl of rehydration solution and stored at −20°C until further use.

### Primers and probe design

Pathogens, targeted genes and primers/probe sets are listed in Table 1. For each pathogen or tick, primers and probes were specifically designed for this study. Each primer or probe set was validated on dilution range of several positive controls (Table 1) and real-time TaqMan PCRs on a LightCycler® 480 (LC480) (Roche Applied Science, Germany). Real-time PCR assays were performed in a final volume of 12 μl using the LightCycler® 480 Probe Master Mix 1× (Roche Applied Science, Germany), with primers and probes at 200 nM and 2 μl of control DNA. Thermal cycling conditions were as follows: 95°C for 5 min, 45 cycles at 95°C for 10 s and 60°C for 15 s and one final cooling cycle at 40°C for 10 s. Four pathogens (*Borrelia valaisiana*, *Francisella tularensis*, *Coxiella burnetii*, and *Theileria annulata*) were targeted by real-time PCRs on two different sequences to improve detection.

**Table 1:** **List of pathogens, tick species, targets, primers/probe sets, and positive controls**.

**Species**	**Target**	**Name**	**Sequence**	**Length (bp)**	**Positive control**
*Borrelia burgdorferi* sensu stricto	*rpoB*	Bo_bu_rpoB_F	GCTTACTCACAAAAGGCGTCTT	83	Culture of B31 strain
		Bo_bu_rpoB_R	GCACATCTCTTACTTCAAATCCT		
		Bo_bu_rpoB_P	AATGCTCTTGGACCAGGAGGACTTTCA		
*Borrelia garinii*	*rpoB*	Bo_ga_rpoB_F	TGGCCGAACTTACCCACAAAA	88	Culture of NE11 strain
		Bo_ga_rpoB_R	ACATCTCTTACTTCAAATCCTGC		
		Bo_ga_rpoB_P	TCTATCTCTTGAAAGTCCCCCTGGTCC		
*Borrelia afzelii*	*fla*	Bo_af_fla_F	GGAGCAAATCAAGATGAAGCAAT	116	Culture of VS641 strain
		Bo_af_fla_R	TGAGCACCCTCTTGAACAGG		
		Bo_af_fla_P	TGCAGCCTGAGCAGCTTGAGCTCC		
*Borrelia valaisiana*	*ospE*	Bo_val_ospE_F	GAAACTTAGGGAGTATCTTATGAAT	143	Culture of VS116 strain
		Bo_val_ospE_R	CTTGCCCCCTTAAACTAATATCT		
		Bo_val_ospE_P	TGCTCACTCAACCTGCCTTGCTCGC		
	*ospA*	Bo_va_ospA_F	ACTCACAAATGACAGATGCTGAA	135	
		Bo_va_ospA_R	GCTTGCTTAAAGTAACAGTACCT		
		Bo_va_ospA_P	TCCGCCTACAAGATTTCCTGGAAGCTT		
*Borrelia miyamotoi*	*glpQ*	B_miya_glpQ_F	CACGACCCAGAAATTGACACA	94	Plasmid^a^
		B_miya_glpQ_R	GTGTGAAGTCAGTGGCGTAAT		
		B_miya_glpQ_P	TCGTCCGTTTTCTCTAGCTCGATTGGG		
*Borrelia spielmanii*	*fla*	Bo_spi_fla_F	ATCTATTTTCTGGTGAGGGAGC	71	Plasmid^a^
		Bo_spi_fla_R	TCCTTCTTGTTGAGCACCTTC		
		Bo_spi_fla_P	TTGAACAGGCGCAGTCTGAGCAGCTT		
*Borrelia lusitaniae*	*rpoB*	Bo_lus_rpoB_F	CGAACTTACTCATAAAAGGCGTC	87	Culture of Poti-B1 strain
		Bo_lus_rpoB_R	TGGACGTCTCTTACTTCAAATCC		
		Bo_lus_rpoB_P	TTAATGCTCTCGGGCCTGGGGGACT		
*Borrelia bissettii*	*rpoB*	Bo_bi_rpoB_F	GCAACCAGTCAGCTTTCACAG	118	Plasmid^a^
		Bo_bi_rpoB_R	CAAATCCTGCCCTATCCCTTG		
		Bo_bi_rpoB_P	AAAGTCCTCCCGGCCCAAGAGCATTAA		
*Borrelia* spp.	23S rRNA	Bo_bu_sl_23S_F	GAGTCTTAAAAGGGCGATTTAGT	73	
		Bo_bu_sl_23S_R	CTTCAGCCTGGCCATAAATAG		
		Bo_bu_sl_23S_P	AGATGTGGTAGACCCGAAGCCGAGT		
*Anaplasma marginale*	*msp1b*	An_ma_msp1_F	CAGGCTTCAAGCGTACAGTG	85	Experimentally infected cow
		An_ma_msp1_R	GATATCTGTGCCTGGCCTTC		
		An_ma_msp1_P	ATGAAAGCCTGGAGATGTTAGACCGAG		
*Anaplasma platys*	*groEL*	An_pla_groEL_F	TTCTGCCGATCCTTGAAAACG	75	Infected dog blood
		An_pla_groEL_R	CTTCTCCTTCTACATCCTCAG		
		An_pla_groEL_P	TTGCTAGATCCGGCAGGCCTCTGC		
*Anaplasma ovis*	*msp4*	An_ov_msp4_F	TCATTCGACATGCGTGAGTCA	92	Plasmid^a^
		An_ov_msp4_R	TTTGCTGGCGCACTCACATC		
		An_ov_msp4_P	AGCAGAGAGACCTCGTATGTTAGAGGC		
*Anaplasma centrale*	*groEL*	An_cen_groEL_F	AGCTGCCCTGCTATACACG	79	Plasmid^a^
		An_cen_groEL_R	GATGTTGATGCCCAATTGCTC		
		An_cen_groEL_P	CTTGCATCTCTAGACGAGGTAAAGGGG		
*Anaplasma phagocytophilum*	*msp2*	An_ph_msp2_F	GCTATGGAAGGCAGTGTTGG	77	Infected embrionary cells of *Ixodes scapularis*
		An_ph_msp2_R	GTCTTGAAGCGCTCGTAACC		
		An_ph_msp2_P	AATCTCAAGCTCAACCCTGGCACCAC		
*Ehrlichia ruminantium*	*dsb*	Eh_ru_dsb_F	CTCAGAGGGTAATAGATTTACTC	107	Culture of Gardel strain
		Eh_ru_dsb_R	GTATGCAATATCTTCAAGCTCAG		
		Eh_ru_dsb_P	ACTACAGGCCAAGCACAAGCAGAAAGA		
*Ehrlichia canis*	*dsb*	Eh_ca_dsb_F	AATACTTGGTGAGTCTTCACTCA	110	Plasmid^a^
		Eh_ca_dsb_R	GTTGCTTGTAATGTAGTGCTGC		
		Eh_ca_dsb_P	AAGTTGCCCAAGCAGCACTAGCTGTAC		
*Ehrlichia chaffeensis*	*dsb*	Eh_ch_dsb_F	TATTGCTAATTACCCTCAAAAAGTC	117	Infected wild *Amblyomma americanum*
		Eh_ch_dsb_R	GAGCTATCCTCAAGTTCAGATTT		
		Eh_ch_dsb_P	ATTGACCTCCTAACTAGAGGGCAAGCA		
*Candidatus* Neoehrlichia mikurensis	*groEL*	Neo_mik_groEL_F	AGAGACATCATTCGCATTTTGGA	96	Infected tick
		Neo_mik_groEL_R	TTCCGGTGTACCATAAGGCTT		
		Neo_mik_groEL_P	AGATGCTGTTGGATGTACTGCTGGACC		
*Rickettsia conorii*	23S-5S ITS	Ri_co_ITS_F	CTCACAAAGTTATCAGGTTAAATAG	118	Culture
		Ri_co_ITS_R	CGATACTCAGCAAAATAATTCTCG		
		Ri_co_ITS_P	CTGGATATCGTGGCAGGGCTACAGTAT		
*Rickettsia slovaca*	23S-5S ITS	Ri_slo_ITS_F	GTATCTACTCACAAAGTTATCAGG	138	Culture
		Ri_slo_ITS_R	CTTAACTTTTACTACAATACTCAGC		
		Ri_slo_ITS_P	TAATTTTCGCTGGATATCGTGGCAGGG		
*Rickettsia massiliae*	23S-5S ITS	Ri_ma_ITS_F	GTTATTGCATCACTAATGTTATACTG	128	Culture
		Ri_ma_ITS_R	GTTAATGTTGTTGCACGACTCAA		
		Ri_ma_ITS_P	TAGCCCCGCCACGATATCTAGCAAAAA		
*Rickettsia helvetica*	23S-5S ITS	Ri_he_ITS_F	AGAACCGTAGCGTACACTTAG	79	Culture
		Ri_he_ITS_R	GAAAACCCTACTTCTAGGGGT		
		Ri_he_ITS_P	TACGTGAGGATTTGAGTACCGGATCGA		
Spotted fever group	*gltA*	SFG_gltA_F	CCTTTTGTAGCTCTTCTCATCC	145	
		SFG_gltA_R	GCGATGGTAGGTATCTTAGCAA		
		SFG_gltA_P	TGGCTATTATGCTTGCGGCTGTCGGT		
*Bartonella henselae*	*pap31*	Bar_he_pap31_F	CCGCTGATCGCATTATGCCT	107	Culture of Berlin 1 strain
		Bar_he_pap31_R	AGCGATTTCTGCATCATCTGCT		
		Bar_he_pap31_P	ATGTTGCTGGTGGTGTTTCCTATGCAC		
*Bartonella quintana*	*bqtR*	Bar_qu_bqt_F	TCCATCACAAGATCTCCGCG	80	Culture
		Bar_qu_bqt_R	CGTGCCAATGCTCGTAACCA		
		Bar_qu_bqt_P	TTTAAGAGAGGAGGTAGAAGAGGCTCC		
*Francisella tularensis*	*tul4*	Fr_tu_tul4_F	ACCCACAAGGAAGTGTAAGATTA	76	Culture of CIP 5612T strain
		Fr_tu_tul4_R	GTAATTGGGAAGCTTGTATCATG		
		Fr_tu_tul4_P	AATGGCAGGCTCCAGAAGGTTCTAAGT		
	*fopA*	Fr_tu_fopA_F	GGCAAATCTAGCAGGTCAAGC	91	
		Fr_tu_fopA_R	CAACACTTGCTTGAACATTTCTAG		
		Fr_tu_fopA_P	AACAGGTGCTTGGGATGTGGGTGGTG		
*Coxiella burnettii*	*idc*	Co_bu_icd_F	AGGCCCGTCCGTTATTTTACG	74	Culture
		Co_bu_icd_R	CGGAAAATCACCATATTCACCTT		
		Co_bu_icd_P	TTCAGGCGTTTTGACCGGGCTTGGC		
	IS*1111*	Co_bu_IS111_F	TGGAGGAGCGAACCATTGGT	86	
		Co_bu_IS111_R	CATACGGTTTGACGTGCTGC		
		Co_bu_IS111_P	ATCGGACGTTTATGGGGATGGGTATCC		
*Babesia divergens*	*hsp70*	Bab_di_hsp70_F	CTCATTGGTGACGCCGCTA	83	Culture of RFS strain
		Bab_di_hsp70_R	CTCCTCCCGATAAGCCTCTT		
		Bab_di_hsp70_P	AGAACCAGGAGGCCCGTAACCCAGA		
*Babesia caballi*	*Rap1*	Ba_cab_rap1_F	GTTGTTCGGCTGGGGCATC	94	Plasmid^a^
		Ba_cab_rap1_R	CAGGCGACTGACGCTGTGT		
		Ba_cab_rap1_P	TCTGTCCCGATGTCAAGGGGCAGGT		
*Babesia canis*	18S rRNA	Ba_ca_RNA18S_F	TGGCCGTTCTTAGTTGGTGG	104	Infected dog blood
		Ba_ca_RNA18S_R	AGAAGCAACCGGAAACTCAAATA		
		Ba_ca_RNA18S_P	ACCGGCACTAGTTAGCAGGTTAAGGTC		
*Babesia vogeli*	*hsp70*	Ba_vo_hsp70_F	TCACTGTGCCTGCGTACTTC	87	Infected dog blood
		Ba_vo_hsp70_R	TGATACGCATGACGTTGAGAC		
		Ba_vo_hsp70_P	AACGACTCCCAGCGCCAGGCCAC		
*Babesia venatorum* (sp. EU1)	18S rRNA	Bab_EU_RNA18S_F	GCGCGCTACACTGATGCATT	91	Plasmid^a^
		Bab_EU_RNA18S_R	CAAAAATCAATCCCCGTCACG		
		Bab_EU_RNA18S_P	CATCGAGTTTAATCCTGTCCCGAAAGG		
*Babesia microti*	*CCTeta*	Bab_mi_CCTeta_F	ACAATGGATTTTCCCCAGCAAAA	145	Culture of R1 strain
		Bab_mi_CCTeta_R	GCGACATTTCGGCAACTTATATA		
		Bab_mi_CCTeta_P	TACTCTGGTGCAATGAGCGTATGGGTA		
*Babesia bovis*	*CCTeta*	Ba_bo_CCTeta_F	GCCAAGTAGTGGTAGACTGTA	100	Culture of MO7 strain
		Ba_bo_CCTeta_R	GCTCCGTCATTGGTTATGGTA		
		Ba_bo_CCTeta_P	TAAAGACAACACTGGGTCCGCGTGG		
*Babesia bigemina*	18S rRNA	Ba_big_RNA18S_F	ATTCCGTTAACGAACGAGACC	99	Plasmid^a^
		Ba_big_RNA18S_R	TTCCCCCACGCTTGAAGCA		
		Ba_big_RNA18S_P	CAGGAGTCCCTCTAAGAAGCAAACGAG		
*Babesia major*	*CCTeta*	Ba_maj_CCTeta_F	CACTGGTGCGCTGATCCAA	75	Plasmid^a^
		Ba_maj_CCTeta_R	TCCTCGAAGCATCCACATGTT		
		Ba_maj_CCTeta_P	AACACTGTCAACGGCATAAGCACCGAT		
*Babesia ovis*	18S rRNA	Ba_ov_RNA18S_F	TCTGTGATGCCCTTAGATGTC	92	Plasmid^a^
		Ba_ov_RNA18S_R	GCTGGTTACCCGCGCCTT		
		Ba_ov_RNA18S_P	TCGGAGCGGGGTCAACTCGATGCAT		
*Theileria equi*	*ema1*	Th_eq_ema1_F	GGCTCCGGCAAGAAGCACA	66	Plasmid^a^
		Th_eq_ema1_R	CTTGCCATCGACGACCTTGA		
		Th_eq_ema1_P	CTTCAAGGCTCCAGGCAAGCGCGT		
*Theileria annulata*	18S rRNA	Th_an_18S_F	GCGGTAATTCCAGCTCCAATA	126	Culture of D7 strain
		Th_an_18S_R	AAACTCCGTCCGAAAAAAGCC		
		Th_an_18S_P	ACATGCACAGACCCCAGAGGGACAC		
	*Tams1*	Th_an_Tams1_F	CGATTACAAACCAGTTGTCGAC	82	
		Th_an_Tams1_R	GTAAAGGACTGATGAGAAGACG		
		Th_an_Tams1_P	TGAGTACTGAGGCGAAGACTGCAAGG		
*Ixodes ricinus*	ITS2	Ix_ri_ITS2_F	CGAAACTCGATGGAGACCTG	77	Tick
		Ix_ri_ITS2_R	ATCTCCAACGCACCGACGT		
		Ix_ri_ITS2_P	TTGTGGAAATCCCGTCGCACGTTGAAC		
*Ixodes persulcatus*	ITS2	Ix_pe_ITS2_F	TGCGTTGCGTCTTCTCTTGTT	111	Tick
		Ix_pe_ITS2_R	TCGATAAAACCAGGTAGGAGGA		
		Ix_pe_ITS2_P	TTTCGGAGCAAGTACAGAGGGAGCAAA		
*Ixodes hexagonus*	ITS2	Ix_hex_ITS2_F	CCGCCGTTGGGATTTACGA	90	Tick
		Ix_hex_ITS2_R	GTTCCTCCGACCCACTTTC		
		Ix_hex_ITS2_P	AGCGCCTTAAAAGAATCGGCAACCTCT		
*Dermacentor reticulatus*	ITS2	De_re_ITS2_F	AACCCTTTTCCGCTCCGTG	83	Tick
		De_re_ITS2_R	TTTTGCTAGAGCTCGACGTAC		
		De_re_ITS2_P	TACGAAGGCAAACAACGCAAACTGCGA		
*Dermacentor marginatus*	ITS2	De_ma_ITS2_F	GCACGTTGCGTTGTTTGCC	139	Tick
		De_ma_ITS2_R	CCGCTCCGCGCAAGAATCT		
		De_ma_ITS2_P	TTCGGAGTACGTCGAGCTCTAGCAGA		
*Escherichia coli*	*eae*	eae-F2	CATTGATCAGGATTTTTCTGGTGATA	102	Culture of EDL933 strain
		eae-R	CTCATGCGGAAATAGCCGTTA		
		eae-P	ATAGTCTCGCCAGTATTCGCCACCAATACC		

a *Plasmids are recombinant pBluescript IISK+ containing the target gene*.

### DNA pre-amplification

For DNA pre-amplification, the TaqMan PreAmp Master Mix (Applied Biosystems, France) was used according to the manufacturer's instructions. Primers (except those which target tick DNA) were pooled combining equal volume of primers (200 nM final each). The reaction was performed in a final volume of 5 μ l containing 2.5 μ l TaqMan PreAmp Master Mix, 1.2 μ l pooled primers mix and 1.3 μ l DNA, with one cycle at 95°C for 10 min, 14 cycles at 95°C for 15 s and 4 min at 60°C. At the end of the cycling program the reactions were diluted 1:10. Pre-amplified DNAs were stored at −20°C until needed.

### High-throughput real-time PCR system

The BioMark™ real-time PCR system (Fluidigm, USA) was used for high-throughput microfluidic real-time PCR amplification using either the 96.96 or the 48.48 dynamic arrays (Fluidigm). These chips dispense 96 (or 48) PCR mixes and 96 (or 48) samples into individual wells, after which on-chip microfluidics assemble PCR reactions in individual chambers prior to thermal cycling resulting in either 9216 or 2304 individual reactions.

Amplifications were performed using 6-carboxyfluorescein (FAM)- and black hole quencher (BHQ1)-labeled TaqMan probes with TaqMan Gene expression master mix in accordance with manufacturer's instructions (Applied Biosystems, France). A 6 μ l sample mix was prepared per sample, containing 3 μl TaqMan® Gene expression Master Mix (Applied Biosystems, Foster City, CA), 0.3 μl sample Loading Reagent (Fluidigm PN 85000746) and 2.7 μl of diluted pre-amplified DNA. A TaqMan® primer assay was prepared for each target, containing 18 μM of each primer and 4 μM of probe. Three microliters of these primer assays were mixed with equal volumes of Dynamic Array (DA) assay loading reagent (Fluidigm PN 85000736) to make assay mixes (9 μ M primers and 2 μ M probe). Prior to loading the samples and assay mixes into the inlets, the chip was primed in the IFC Controller HX apparatus. Five μl of sample mixes, prepared as described, were then loaded into each sample inlet of the dynamic array chip and 5 μ l of assay mixes were loaded into assay inlets. The chip was then placed on the IFC Controller HX for loading and mixing. After approximately 45 min the chip was ready for thermal cycling and detection of the reaction products on the Biomark. PCR cycling comprised of 2 min at 50°C, 10 min at 95°C, followed by 40 cycles of 2-step amplification of 15 s at 95°C, and 1 min at 60°C. Data were acquired on the BioMark™ Real-Time PCR System and analyzed using the Fluidigm Real-time PCR Analysis software to obtain crossing point (CP) values.

For microfluidic tool evaluation on field samples, the assays were performed in duplicate. Two negative water controls were included per chip. *Ixodes ricinus* DNA served to confirm the tested tick species and as a DNA extraction control. To determine if factors present in the sample could inhibit the PCR, *Escherichia coli* strain EDL933 DNA was added to each sample as an internal inhibition control. Primers and probe specific for the *E. coli eae* gene (Nielsen and Andersen, 2003) were used for an internal control.

### Validation of the results by PCR and sequencing

Conventional PCR using primers targeting different genes or regions than those of the BioMark™ system (Table 2), were used to confirm the presence of pathogenic DNA in the field samples. Amplicons were sequenced by Eurofins MWG Operon (Germany), and then assembled using BioEdit software (Ibis Biosciences, Carlsbad). An online BLAST (National Center for Biotechnology Information) was used to compare results with published sequences listed in GenBank sequence databases.

**Table 2:** **Primers used to confirm the presence of pathogenic DNA in ticks**.

**Pathogen**	**Targeted gene**	**Primer name**	**Sequence (5′ → 3′)**	**Amplicon size (bp)**	**References**
*Borrelia* spp.	*clpA*	clpAF1240	GATAGATTTCTTCCAGACAAAG	975	Margos et al., 2008
		clpAR2214	TTCATCTATTAAAAGCTTTCCC		
		clpAF1255	GACAAAGCTTTTGATATTTTAG	850	
		clpAR2104	CAAAAAAAACATCAAATTTTCTATCTC		
*Bartonella* spp.	16S-23S IGS	P-bhenfa	TCTTCGTTTCTCTTTCTTCA	186	Rampersad et al., 2005
		P-benr1	CAAGCGCGCGCTCTAACC		
		N-bhenf1a	GATGATCCCAAGCCTTCTGGC	149	
		N-benr	AACCAACTGAGCTACAAGCC		
*Anaplasma phagocytophilum*	*msp4*	MSP4AP5	ATGAATTACAGAGAATTGCTTGTAGG	849	De La Fuente et al., 2005
		MSP4AP3	TTAATTGAAAGCAAATCTTGCTCCTATG		
*Candidatus* N. mikurensis	*groEL*	NM 1152as	TTCTACTTTGAACATTTGAAGAATTACTAT	1024	Diniz et al., 2011
		NM 128s	AACAGGTGAAACACTAGATAAGTCCAT		
*Rickettsia* spp.	*gltA*	Rsfg877	GGGGGCCTGCTCACGGCGG	381	Regnery et al., 1991
		Rsfg1258	ATTGCAAAAAGTACAGTGAACA		
*Babesia* spp.	18S rRNA	BabGF2	GYYTTGTAATTGGAATGATGG	559	Bonnet et al., 2007b
		BabGR2	CCAAAGACTTTGATTTCTCTC		

### Prevalence estimation

Prevalences were estimated assuming perfect sensitivity and specificity of pathogen detection using the online statistical program “Pooled prevalence for fixed pool size and perfect test” Method 2 (AusVet Animal Health Service http://epitools.ausvet.com.au/content.php?page=home). Point estimates were based on the maximum likelihood method developed by Kline et al. (1989). Exact 95% confidence intervals were obtained by assuming binomial distribution for the number of positive pools (Cowling et al., 1999). If all pools were positive, prevalence was recorded as >14.3%, as the highest prevalence that can be distinguished from 100% when testing 47 pools of 25 ticks. If all pools were negative, prevalence was recorded as <0.25%, since the 95% probability of sampling *n* negative ticks from a population with prevalence *p* is given as (1 − *p*)^*n*^.

## Results

### Implementation of high-throughput real-time PCR system to detect TBPs

**Figure 1:**
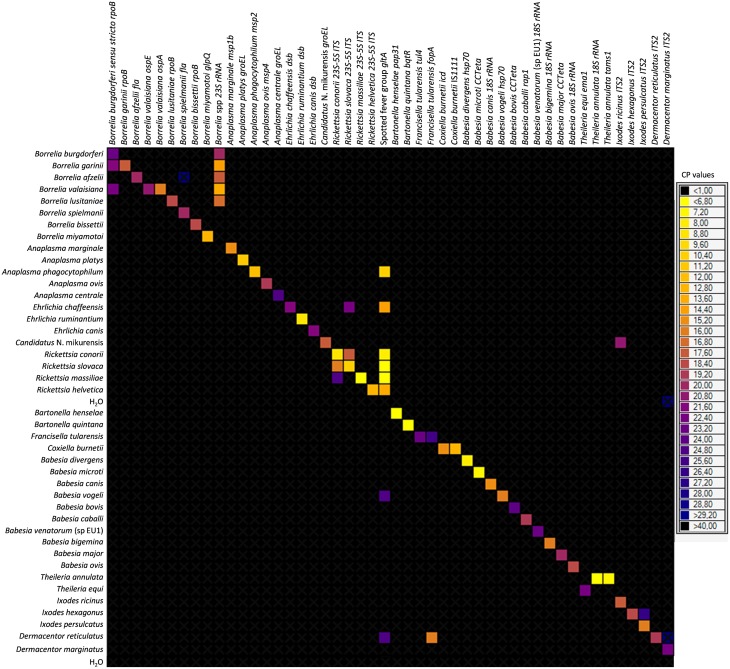
**BioMark™ dynamic array system specificity test (48.48 chip)**. Each square corresponds to a single real-time PCR reaction, where rows indicate the pathogen in the positive control and columns represent the targets of each primers/probe set. CP values for each reaction are indicated by color; the corresponding color scale is presented in the legend on the right. The darkest shade of blue and black squares are considered as negative reactions with *CP* > 30.

Primers and probes were specially designed to detect 37 TBPs and 4 tick species (Table 1). Each set of primers and probes specifically identified their corresponding positive control samples via Taqman real-time PCRs on a LightCycler 480 apparatus. Resulting CP values varied from 8 to 40 depending on sample type. Among the 37 TBP DNAs used as positive controls, 10 were not detected by the BioMark™ system. Consequently, an initial step of DNA pre-amplification was added, which enabled detection of all positive controls. Subsequently all tick DNA samples were pre-amplified prior to pathogen detection on the BioMark™ system. The specificity of each primer set was then evaluated using 37 TBPs, and 4 tick species positive controls (Figure 1). Results demonstrated high specificity for each primers/probe set after pre-amplification, using a cut-off of 30 CP (Figure 1). Indeed, 45 assays were only positive for the corresponding positive control. Three assays showed cross-reactivity with other pathogen targets. The assay for *B. burgdorferi* sensu stricto cross-reacted with *B. garinii* and *B. valaisiana* DNA. The assay targeting *R. conorii* cross-reacted with *R. massiliae*, as well as with *R. slovaca* DNA, cross-reactivity was also observed reciprocally.

### Large scale prevalence study of TBPs

A total of 7050 nymphs, in 47 pools of 25, from six different European sites were tested using the BioMark™ system. Among the targeted pathogens, 15 bacteria (*B. lusitaniae*, *B. bissettii*, *A. marginale*, *A. platys*, *A. ovis*, *A. centrale, E. ruminantium*, *E. canis*, *E. chaffeensis, R. conorii*, *R. slovaca*, *R. massiliae, B. quintana, F. tularensis*, and *C. burnetii*) and 10 parasites (*B. caballi*, *B. canis*, *B. vogeli*, *B. microti*, *B. bovis*, *B. bigemina*, *B. major*, *B. ovis*, *T. equi*, and *T. annulata*) were not detected in any country. The number of positive pools for each pathogen is presented in Table 3 and the prevalence was estimated at each site of collection (Table 4).

**Table 3:** **Number of positive pools of ticks out of the 47 tested, for two sites in France, Denmark, and the Netherlands using the microfluidic tool (BioMark™ system)**.

	**Number of positive pools (out of 47 tested)**					
	**France**		**Denmark**		**The Netherlands**	
	**Murbach F1**	**Wasselonne F2**	**Vestskoven D1**	**Grib Skov D2**	**Duin en Kruidberg N1**	**Austerlitz N2**
*Borrelia* spp.	**32**	**33**	**47**	**40**	**38**	**44**
*B. burgdorferi* sensu stricto	**0**	**1**	**31**	**29**	**14**	**6**
*B. garinii*	**5**	**8**	**19**	**36**	**20**	**17**
*B. afzelii*	**13**	**17**	**46**	**32**	**20**	**36**
*B. valaisiana*	**1**	**1**	**13**	**11**	**6**	**1**
*B. spielmanii*	**1**	**1**	**10**	**17**	**3**	**1**
*B. miyamotoi*	**22**	**10**	**13**	**2**	**20**	**27**
*B. lusitaniae*	0	0	0	0	0	0
*B. bissettii*	0	0	0	0	0	0
*Anaplasma marginale*	0	0	0	0	0	0
*Anaplasma platys*	0	0	0	0	0	0
*Anaplasma ovis*	0	0	0	0	0	0
*Anaplasma centrale*	0	0	0	0	0	0
*Anaplasma phagocytophilum*	**8**	**12**	**4**	**45**	**10**	**19**
*Ehrlichia ruminantium*	0	0	0	0	0	0
*Ehrlichia canis*	0	0	0	0	0	0
*Ehrlichia chaffeensis*	0	0	0	0	0	0
*Candidatus* N. mikurensis	**13**	**2**	**10**	**2**	**28**	**41**
Spotted fever group	**46**	**46**	**44**	**47**	**45**	**32**
*Rickettsia conorii*	0	0	0	0	0	0
*Rickettsia slovaca*	0	0	0	0	0	0
*Rickettsia massiliae*	0	0	0	0	0	0
*Rickettsia helvetica*	**46**	**46**	**44**	**46**	**45**	**32**
*Bartonella henselae*	**0**	**1**	**0**	**0**	**0**	**0**
*Bartonella quintana*	0	0	0	0	0	0
*Francisella tularensis*	0	0	0	0	0	0
*Coxiella burnetii*	0	0	0	0	0	0
*Babesia divergens*	**0**	**0**	**1**	**1**	**0**	**2**
*Babesia caballi*	0	0	0	0	0	0
*Babesia canis*	0	0	0	0	0	0
*Babesia vogeli*	0	0	0	0	0	0
*Babesia venatorum* (sp. EU1)	**2**	**3**	**14**	**5**	**0**	**9**
*Babesia microti*	0	0	0	0	0	0
*Babesia bovis*	0	0	0	0	0	0
*Babesia bigemina*	0	0	0	0	0	0
*Babesia major*	0	0	0	0	0	0
*Babesia ovis*	0	0	0	0	0	0
*Theileria equi*	0	0	0	0	0	0
*Theileria annulata*	0	0	0	0	0	0

*Bold values represent pathogens detected at least in one site*.

**Table 4:** **Estimated prevalence of pathogens detected in *Ixodes ricinus* in France, Denmark, and the Netherlands**.

	**Estimated prevalence % (95% CI)**					
	**France**		**Denmark**		**The Netherlands**	
	**Murbach F1**	**Wasselonne F2**	**Vestskoven D1**	**Grib Skov D2**	**Duin en Kruidberg N1**	**Austerlitz N2**
*Borrelia* spp.	4.47 (2.97–6.41)	4.73 (3.15–6.77)	>14.27^b^	7.33 (4.92–10.52)	6.40 (4.31–9.12)	10.42 (6.73–15.85)
*B. burgdorferi* sensu stricto	<0.25^a^	0.09 (0.00–0.48)	4.22 (2.79–6.08)	3.77 (2.46–5.47)	1.40 (0.76–2.36)	0.54 (0.20–1.18)
*B. garinii*	0.45 (0.14–1.05)	0.74 (0.32–1.46)	2.05 (1.22–3.21)	5.64 (3.79–8.04)	2.19 (1.32–3.39)	1.78 (1.02–2.85)
*B. afzelii*	1.29 (0.68–2.20)	1.78 (1.02–2.85)	14.27 (8.35–26.0)	4.47 (2.97–6.41)	2.19 (1.32–3.39)	5.64 (3.79–8.04)
*B. valaisiana*	0.09 (0.00–0.48)	0.09 (0.00–0.48)	1.29 (0.68–2.20)	1.06 (0.52–1.90)	0.54 (0.20–1.18)	0.09 (0.00–0.48)
*B. spielmanii*	0.09 (0.00–0.48)	0.09 (0.00–0.48)	0.95 (0.45–1.75)	1.78 (1.02–2.85)	0.26 (0.05–0.77)	0.09 (0.00–0.48)
*B. miyamotoi*	2.49 (1.54–3.79)	0.95 (0.45–1.75)	1.29 (0.68–2.20)	0.17 (0.02–0.63)	2.19 (1.32–3.39)	3.36 (2.17–4.93)
*Anaplasma phagocytophilum*	0.74 (0.32–1.46)	1.17 (0.60–2.05)	0.36 (0.10–0.91)	11.86 (7.42–18.97)	0.95 (0.45–1.75)	2.05 (1.22–3.21)
*Candidatus* N. mikurensis	1.29 (0.68–2.20)	0.17 (0.02–0.63)	0.95 (0.45–1.75)	0.17 (0.02–0.63)	3.56 (2.31–5.19)	7.90 (5.28–11.41)
Spotted fever group	14.27 (8.35–26.0)	14.27 (8.35–26.0)	10.42 (6.73–15.85)	>14.27^b^	11.86 (7.42–18.97)	4.47 (2.97–6.41)
*Rickettsia helvetica*	14.27 (8.35–26.0)	14.27 (8.35–26.0)	10.42 (6.73–15.85)	14.27 (8.35–26.0)	11.86 (7.42–18.97)	4.47 (2.97–6.41)
*Bartonella henselae*	<0.25^a^	0.09 (0.00–0.48)	<0.25^a^	<0.25^a^	<0.25^a^	<0.25^a^
*Babesia divergens*	<0.25^a^	<0.25^a^	0.09 (0.00–0.48)	0.09 (0.00–0.48)	<0.25^a^	0.17 (0.02–0.63)
*Babesia venatorum* (sp. EU1)	0.17 (0.02–0.63)	0.26 (0.05–0.77)	1.40 (0.76–2.36)	0.45 (0.14–1.05)	<0.25^a^	0.85 (0.38–1.60)

a All pools negative;

b *all pools positive*.

*Point estimates were based on the maximum likelihood method developed by Kline et al. (16). If all pools were positive, prevalence was recorded as >14.3%, as the highest prevalence that can be distinguished from 100% when testing 47 pools of 25 ticks. If all pools were negative, prevalence was recorded as <0.25%, since the 95% probability of sampling n negative ticks from a population with prevalence p is given as (1−p)n*.

In order to confirm the results obtained on the BioMark™ system and to validate this new method, classical PCR and sequencing were performed on extracted DNA for a subset of field samples. All sequences showed at least 99% identity with reference sequences (Table 5), and have been deposited in GenBank (Accession numbers; KF447526-KF447532, and KF679796). Due to primers which can only detect *Borrelia* and *Babesia* at the genus level, only those samples which tested positive for a single species (and not potentially co-infected samples) were confirmed (Table 2).

**Table 5:** **Homology between deposited sequences and reference sequences in GenBank**.

**Species**	**Nb of samples tested**	**Nb of samples obtained after sequencing**	**Deposited sequence**	**Length (bp)**	**Percentage of identity (%)**	**Reference sequence**
*Borrelia garinii*	1	1	KF447529	822	99	AB555782
*Borrelia afzelii*	1	1	KF447528	824	99	JX971251
*Bartonella henselae*	1	1	KF679796	149	100	FJ832091
*Anaplasma phagocytophilum*	12	3	KF447526	824	100	EF067343
*Candidatus* N. mikurensis	12	8	KF447527	1012	100	EU810407
*Rickettsia helvetica*	12	9	KF447530	382	100	JX040636
*Babesia divergens*	3	2	KF447531	527	99	AY572456
*Babesia venatorum* (sp. EU1)	13	10	KF447532	562	100	JQ993425

#### France

Among the seven genospecies of *Borrelia burgdorferi* s.l., four were detected in both French sites. *Borrelia afzelii* is the dominant genospecies with a prevalence of 1.8% in F2, as previously described (Beytout et al., 2007). The other genospecies (*B. garinii*, *B. valaisiana*, and *B. spielmanii*) had prevalence rates of under 1%. *B. burgdorferi* s.s. was only detected in F2 at low prevalence (0.1%). The relapsing fever spirochete *B. miyamotoi* was detected in both sites, with very different prevalences (2.5% in F1 and 0.9% in F2) and was the most abundant *Borrelia* species in F1. This spirochete has already been detected in France, but only in female adult ticks (Reis et al., 2011). *Anaplasma phagocytophilum* was more abundant in F2 (1.2%) and its estimated prevalence is in accordance with a previous study (Beytout et al., 2007). *Candidatus* N. mikurensis was more abundant in F1 (1.3%). This pathogen has already been described in bank voles in France (Vayssier-Taussat et al., 2012) but this is the first estimation of its prevalence in French ticks. *Rickettsia helvetica* was the only *Rickettsiaceae* identified in this study and was detected in 46/47 pools, showing the highest prevalence (14.3%) of all tested pathogens, much higher than data reported in the literature (1.4–6%) (Cotte et al., 2010). *Bartonella henselae* was only detected in F2 (0.1%), in a single pool. Among all assessed parasitic species, *Babesia venatorum* was the only parasite detected in France with a low prevalence (0.2 and 0.3%) as previously described (Reis et al., 2011).

#### Denmark

Five genospecies of *B. burgdorferi* s.l. were detected in Danish ticks, four previously described (Skarphedinsson et al., 2007; Vennestrom et al., 2008) and one, *B. spielmanii*, detected for the first time. In previous studies, *B. afzelii* was the most prevalent genospecies (Skarphedinsson et al., 2007; Vennestrom et al., 2008). In our study, *B. afzelii* was the most prevalent (14.3%) in D1, while *B. garinii* was the most abundant (5.7%) in D2. *B. burgdorferi* s.s. was detected in both sites with similar prevalences (4.2% and 3.8%), as well as *B. valaisiana*, and *B. spielmanii* (approximately 1%). *B. lusitaniae* was identified in a previous study (Vennestrom et al., 2008), but was not encountered in the present study. Relapsing fever-causing *B. miyamotoi* was detected for the first time in Danish *I. ricinus* with variable prevalences between the two sites (1.3% in D1 and 0.2% in D2). The estimated prevalence of *A. phagocytophilum* was approximately 30 times higher in D2 (11.9%) than in D1 (0.4%) whereas its prevalence was estimated at 15% in a previous study (Skarphedinsson et al., 2007). *Candidatus* N. mikurensis was detected with a low prevalence in the Danish sites (1% in D1 and 0.2% in D2) in agreement with a previous report (Fertner et al., 2012). *Rickettsia helvetica* is the only species of *Rickettsia* spp. reported in Denmark. This bacterium was respectively identified in 44 and 46 pools of the samples, corresponding to high prevalences of 10.4% in D1 and 14.3% in D2. In previous reports, the prevalence of *R. helvetica* appeared to vary considerably, ranging from 1.4 to 13% (Svendsen et al., 2009; Kantso et al., 2010). Two parasitic species were found for the first time in the Danish samples, *B. divergens* (0.1% in D1 and D2) and *B. venatorum* (1.4% in D1 and 0.5% in D2). These parasites have never previously been reported in Danish ticks until now, even if *B. divergens* is frequently found in cattle.

#### The Netherlands

Five genospecies of *B. burgdorferi* s.l. were detected in Dutch ticks. *B. garinii* and *B. afzelii* were the more abundant genospecies while the other genospecies (*B. burgdorferi*, *B. valaisiana*, and *B. spielmanii*) were found less frequently, as previously described (Tijsse-Klasen et al., 2011; Sprong et al., 2012a). *B. garinii* and *B. afzelii* were detected with equal prevalences in N1 (2.2%) and variable prevalences in N2 (1.8 and 5.6%, respectively). The prevalence of *B. burgdorferi* s.s. was estimated at 1.4 and 0.5% in N1 and N2, respectively. *B. valaisiana* and *B. spielmanii* were identified in a single pool from the N2 site, but their prevalences were estimated at 0.5 and 0.3% in N1. In 2009, *B. lusitaniae* was described at one location (Sprong et al., 2012a), but was not encountered in the present study. The relapsing fever spirochete, *B. miyamotoi*, previously identified in the Netherlands in a human case of meningoencephalitis (Hovius et al., 2013), occurred in both Dutch sites and was most prevalent in N2 (3.4%). In N1, *B. miyamotoi* showed the same prevalence as *B. garinii* and *B. afzelii* (2.2%). *Anaplasma phagocytophilum* and *Candidatu*s N. mikurensis were found in both sites with variable prevalences, both more abundant in N2 (2 and 7.9%, respectively). The estimated prevalence of *R. helvetica* was highly variable depending on the sites (11.9% in N1 and 4.5% in N2). These three bacteria are well recognized in Dutch ticks and have previously been reported in the Netherlands (Nijhof et al., 2007; Sprong et al., 2009; Tijsse-Klasen et al., 2011). Two parasitic species were found in Dutch ticks but were only observed in N2, *B. divergens* (0.2%) and *B. venatorum* (0.8%) with prevalence rates similar to previous reports (0.07 and 0.4% for *B. divergens* and 0.9 and 1.2% for *B. venatorum*) (Nijhof et al., 2007; Wielinga et al., 2009).

## Discussion

In this study, we implemented a method using multiple primers/probe sets able to perform high-throughput detection of TBPs on an unprecedented scale. This large-scale investigation has (i) enabled the detection of rare pathogens such as *Bartonella henselae* and (ii) generated prevalence estimations for frequent, rare, or unexpected pathogens, thus creating a comprehensive overview of the epidemiological situation for 37 bacteria and parasites present in *I. ricinus*, in six European sites (two in France, Denmark, and the Netherlands).

Initial testing of the BioMark™ system showed that some pathogens could not be detected. Indeed, assessment was performed on positive DNA controls extracted from cultures, animal blood, ticks, or plasmids, therefore DNA quality and concentration were highly variable between samples. An initial step of pre-amplification was therefore added to specifically amplify targeted pathogen sequences. Regarding the three non-specific assays, two hypotheses can be made: either lack of specificity or potential co-infection of the DNA samples. As positive controls were isolated from pure bacterial cultures, only non-specific cross-reaction explains the lack of specificity. The set of primers and probe designed against *B. burgdorferi* s.s. cross-reacted with *B. garinii* and *B. valaisiana*. However, this cross-reaction did not occur for every field sample. Cross-reactions were also observed between *R. conorii* and *R. slovaca*. There was a difference of approximately 10 cycles between the CP values for the expected *Rickettsia* species and the cross-reacting species. It will be interesting to test both sets of primers and probe on DNA extracted from ticks uniquely infected with each of the *Rickettsia*. However, this issue is not likely to arise with field samples, as *R. slovaca* is transmitted by *Dermacentor marginatus* and *R. conorii* by *Rhipicephalus sanguineus*. In conclusion, the primers and probe sets for *B. burgdorferi* s.s., *R. conorii*, and *R. slovaca* need further optimization, so the current results obtained for these species should be interpreted with care. Several of the targeted pathogens cannot be cultured, or are rare and consequently unavailable from field samples, therefore plasmids containing target sequences were used as positive controls. For these pathogens and associated primers/probe sets, further evaluation of specificity is required. This tool was developed for epidemiologic rather than diagnostic purposes, therefore detection limits and sensitivity have not been experimentally determined. These experiments are somewhat difficult to implement and require a gold standard for each pathogen and consistent positive controls, which are not available for all TBPs.

Two sites per country were studied for the field investigation. The technique permitted the detection of 10 bacterial species; *B. burgdorferi* s.s., *B. garinii*, *B. afzelii*, *B. valaisiana*, *B. spielmanii*, *B. miyamotoi*, *A. phagocytophilum*, *Candidatus* N. mikurensis, *R. helvetica*, *B. henselae*, and two parasitic species; *B. divergens*, and *B. venatorum*, with variable prevalences according to the site of collection. Taken together, the estimated prevalences for all pathogens obtained on pools of 25 nymphs in this study are mostly consistent with European published data. For future studies, it will be fascinating to investigate smaller nymph pools to obtain more accurate estimations of TBP prevalences. The prevalence of *B. miyamotoi* is reported for the first time in Denmark at two sites and is quite similar between the three European countries in our study. *Borrelia miyamotoi* is transmitted by the same *Ixodes* species as the etiologic agents of European Lyme borreliosis, and has been detected in *Ixodes* ticks in Europe (Richter et al., 2003). Up until now no human cases have been reported in France or Denmark, but our data and the recent case of human infection described in the Netherlands (Hovius et al., 2013) suggest that surveillance needs to be improved. *Candidatus* N. mikurensis was detected in all three countries, with the highest prevalence in the Netherlands. Several human cases have been reported over the past decade in Europe (Maurer et al., 2013). However, clinical symptoms are not pathognomonic, suggesting the existence of unreported cases due to reduced awareness of symptoms by public health professionals (Jahfari et al., 2012). As this emerging human pathogen is widespread in Europe, it requires careful monitoring. *Rickettsia helvetica* was described as the most prevalent pathogen in all three countries. Even if its pathogenicity remains unclear, *R. helvetica* has been implicated in the development of fatal perimyocarditis (Sprong et al., 2009). Isolation of the bacterium from a patient is needed to definitely confirm *R. helvetica* as a human pathogen; however, *R. helvetica* already represents an excellent candidate for future emergence (Parola, 2004). Over the last few years, *I. ricinus* has been identified as a competent vector for *Bartonella henselae* (Cotte et al., 2008). Little data are available on its prevalence in ticks; and it has been estimated at between 11 and 40% in Europe (Dietrich et al., 2010). *Bartonella henselae* has never been reported in Danish ticks, but two variant types were detected in cats and mice (Engbaek and Lawson, 2004). Its presence in French ticks could be linked to the presence of wild cats in eastern France compared to the other countries. Babesiosis can be a variable but potentially severe disease, and is best known as an animal affliction. However, increasing numbers of human cases have refocused epidemiological attention on this emerging zoonosis (Hildebrandt et al., 2013). Our study demonstrates that these hemoparasites are widely present in European ticks, and were observed for the first time in Danish ticks. *Babesia microti* was not encountered in this study but has previously been detected in the Netherlands at a prevalence ranging from 0.1 to 9% (Wielinga et al., 2009; Tijsse-Klasen et al., 2011).

Interestingly, among the targeted pathogens, 15 bacterial species and 10 parasitic species were not detected in any country, leading us to conclude that they are not present in *I. ricinus* from those European sites. Indeed, these TBPs are either very rare (Parola and Raoult, 2001; Sprong et al., 2012b) or have never been previously detected in the sampled regions, or are transmitted by other stage or other tick species (Parola and Raoult, 2001). *Francisella tularensis* and *Coxiella burnetii* are linked to important human and veterinary public health problems that require surveillance (Sprong et al., 2012b; Carvalho et al., 2014); however, the role of ticks in the transmission of these pathogens is nonetheless debated. Their apparent absence across the three European countries in *I. ricinus* ticks suggests that the risk of acquiring tularemia or Q fever from questing ticks could be negligible.

This new screening approach based on microfluidic systems allowing multiple parallel real-time PCRs, is a powerful tool for TBP surveillance in Europe. This study demonstrates the technique's capacity for large-scale studies utilizing the unique ability to simultaneously analyze large numbers of samples and multiple target pathogens. As demonstrated for babesiosis, vector surveillance could be very useful for monitoring disease emergence (Diuk-Wasser et al., 2014). Compared to an array with fixed panels of probes, this new tool presents the major advantage that it can be easily adapted to new situations, as it is entirely possible to add or remove primers/probe sets in order to modify the panel of targeted pathogens and tick species. Further studies will indeed confirm if this approach heralds the necessary breakthrough in epidemiological surveillance of vector-borne pathogens, broadening the monitoring of human and animal diseases.

In conclusion, our study clearly demonstrates the utility of a fast tool that allows comprehensive testing of high numbers of TBPs in ticks, and can be easily customized to fit regional demands or to screen tick or host samples for new or emerging diseases.

### Conflict of interest statement

The authors declare that the research was conducted in the absence of any commercial or financial relationships that could be construed as a potential conflict of interest.
